# Neurodegeneration as an evolutionary trade-off: a biological constraint–aware therapeutic perspective

**DOI:** 10.1186/s13024-026-00958-w

**Published:** 2026-05-25

**Authors:** Seung Hyun Kim, Min-Young Noh, Matthew C. Kiernan

**Affiliations:** 1 Department of Neurology, College of Medicine, Hanyang University, 222, Wangsimni-ro, Seongdong-gu, Seoul, 04763 Republic of Korea; 2 Cell Therapy Center, Hanyang University Hospital, Seoul, Republic of Korea; 3 Neuroscience Research Australia, Barker St, Randwick, Sydney, NSW 2031 Australia; 4 University of New South Wales, Sydney, Australia

**Keywords:** Neurodegenerative diseases, Evolutionary trade-offs, Antagonistic pleiotropy, Biological constraint, Resilience exhaustion, Selective vulnerability, Therapeutic paradigms

Neurodegenerative diseases (NDDs) have traditionally been viewed as disorders driven by toxic protein aggregation and selective neuronal vulnerability. Although this pathology-centered framework has guided major mechanistic discoveries, interventions aimed at removing aggregates or suppressing downstream pathways have yielded limited and variable clinical benefits [1, 2], suggesting that broader biological contexts remain insufficiently addressed. Here, we propose a biological constraint–aware perspective in which neurodegeneration arises, in part, from the long-term consequences of evolutionary trade-offs and from biological constraints that may also operate independently within highly specialized neural systems. Programs selected to optimize early-life survival, reproduction, immune responsiveness, or neural performance may generate or amplify persistent structural and functional limitations. In parallel, aging, cumulative stress, genetic mutations, exposomal burden, or disease-related pathology may further erode compensatory and buffering capacity. These converging pressures may lead to resilience exhaustion, a state in which adaptive mechanisms become insufficient, and degeneration may emerge once critical thresholds are exceeded.

Central to this view is antagonistic pleiotropy (AP), first articulated by Williams [3], whereby genetic variants that confer early-life fitness advantages may exert deleterious effects later in life because their early benefits outweigh late costs under selection [4]. In the nervous system, such programs may support development, plasticity, and immune responsiveness while imposing long-term proteostatic, metabolic, and inflammatory liabilities. When these programs persist beyond their adaptive window, they may become biological constraints that cannot be selectively silenced without compromising viability. *APOE ε4* provides one candidate example, as it has been proposed to confer context-dependent advantages in immune responsiveness, infection resistance, or metabolic adaptation, while increasing late-life neurodegenerative vulnerability. Similar antagonistic pleiotropy-like patterns have been proposed in repeat expansion disorders, including SBMA and Huntington’s disease, where expanded CAG repeats encoding polyglutamine tracts may confer context-dependent effects on transcriptional regulation, protein interactions, or developmental and reproductive biology, while later increasing proteostatic stress, aggregation propensity, and neuronal vulnerability. Beyond these candidate AP or AP-like examples, other genetic factors may operate within the biological constraint framework by modifying the threshold for resilience exhaustion. Highly penetrant mutations in familial neurodegenerative diseases may instead act as threshold-lowering factors or constraint accelerators within already vulnerable systems. Mutations in *APP*, *MAPT*, *TARDBP*, or *SNCA* may intensify proteostatic, transport, or RNA metabolic burdens in post-mitotic neurons, accelerating transition toward resilience exhaustion. Longevity-associated genetic factors, in contrast, may modulate these trajectories through context-dependent effects on resilience and lifespan, either enhancing maintenance capacity or extending the time window during which late-life vulnerability may emerge.

As post-mitotic cells, neurons lack the capacity for renewal through cell division and therefore rely on highly efficient protein quality control (PQC) systems to maintain homeostasis over decades [5]. Evolutionarily selected programs that optimize early-life neuronal performance and adaptability require sustained proteome maintenance, dynamic RNA metabolism and granule regulation, efficient long-distance transport, and stress-responsive phase transitions over prolonged periods. These systems—including autophagy–lysosomal pathways, proteasomal degradation, RNA-binding protein dynamics, and primary cilia–mediated signaling—enable neurons to cope with metabolic stress, proteostatic load, immune activation, and cumulative molecular damage for extended periods. However, while advantageous early in life, these demands may impose cumulative proteostatic burden with aging and stress, thereby increasing vulnerability to impaired proteostasis, maladaptive protein aggregation, and proteinopathy. As compensatory capacity is progressively depleted over time, buffering mechanisms may fall below a critical threshold, at which point hallmark features such as toxic protein accumulation and neuroinflammation become evident. Cellular senescence may initially function adaptive stress-response program, but in aged neural tissues, persistent senescence and senescence-associated inflammatory burden may become a biological constraint that further erodes systemic resilience. In this sense, NDDs may be understood as exhausted states of systemic resilience operating under fixed biological constraints.

Amyotrophic lateral sclerosis (ALS) provides a clear example of a constraint-driven disorder affecting motor networks [6]. The motor system reflects an evolutionary compromise that prioritizes speed, precision, and long-range connectivity with limited redundancy. These features confer early-life functional advantages but impose exceptional metabolic and proteostatic demands on motor neurons, among the largest and most polarized cells in humans. Consistent with this vulnerability, ALS-associated genetic variants are enriched in pathways related to oxidative stress response, protein clearance, and DNA–RNA binding proteins [7]. By contrast, higher-order cognitive systems evolved with greater redundancy and distributed processing, alongside greater reliance on glial support, lipid metabolism, and immune modulation. Consistent with this architecture, Alzheimer’s disease is enriched for genetic risk loci related to lipid handling and innate immune regulation, including *APOE* and *TREM2*, underscoring the contribution of lipid–immune–glial interactions to late-life neurodegenerative vulnerability [8].

This contrast highlights that neurodegenerative vulnerability is shaped not only by neuronal specialization, but also by glial and immune-related buffering systems. Astrocytes play primary roles in metabolic homeostasis, substrate support, and synaptic regulation, whereas microglia contribute to immune surveillance, debris clearance, metabolic sensing, and proteostatic stress responses. Within this multicellular buffering system, microglia represent one important modulatory component, influencing whether adaptive stress-response programs remain protective or shift toward pathological constraint [9]. Maintaining glial activity within an appropriate functional range is essential: impaired clearance or support may weaken stress adaptation, whereas chronic activation may amplify inflammatory and metabolic burden.

These considerations motivate a shift toward a biological constraint–aware therapeutic strategy (Fig. 1). Rather than indiscriminate pathology removal, this approach emphasizes proportional modulation of immune–glial interactions and stress-response/proteostatic pathways, buffering of metabolic and inflammatory burden, and reinforcement of residual adaptive capacity within constrained neural systems.

Because pharmacological tools capable of safely and precisely modulating these constraints remain under development, non-pharmacological interventions deserve particular emphasis [10]. Tailored physical activity, management of modifiable risk factors, dietary patterns supporting antioxidant balance, sleep and circadian optimization, and reduction of chronic inflammatory burden may help preserve neural resilience. In asymptomatic individuals with strong genetic or familial risk, structured surveillance and anticipatory guidance may further support long-term neural function. Similar principles may also extend beyond the central nervous system, particularly to peripheral tissues and systemic immune and metabolic regulation.

NDDs may therefore reflect the biological costs of evolutionary trade-offs in neural systems built on long-lived, post-mitotic cells. Evolution has selected for survival, adaptability, and function—not unlimited longevity. A constraint-aware therapeutic strategy operates within this reality, aiming not only to reduce pathological burden but also to preserve resilience, restore balance, and extend functional capacity.

**Fig. 1:**
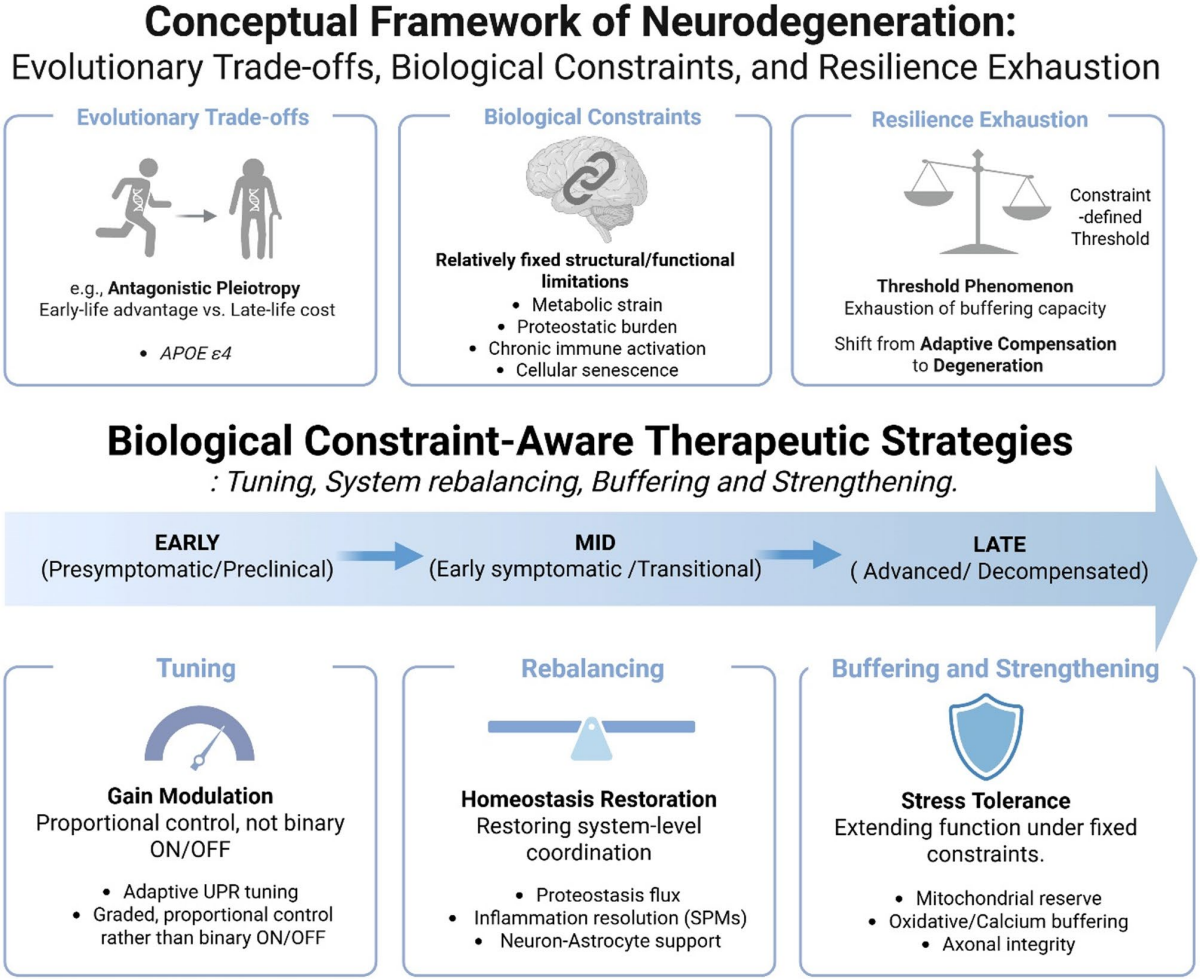
Conceptual framework of neurodegeneration and biological constraint–aware therapeutic strategies

Evolutionary trade-offs, biological constraints, and resilience exhaustion are depicted as distinct but interacting and partially independent contributors to neurodegeneration. Under aging, cumulative stress, genetic factors, and disease-related pathological burden, these converging pressures may exceed the resilience capacity of vulnerable neural systems and promote a shift from adaptive compensation to degeneration.

Upper Panel (Conceptual Framework): Evolutionary trade-offs, including antagonistic pleiotropy, favor early-life survival, performance, or adaptability despite latent late-life costs. These adaptive programs may give rise to relatively fixed biological constraints, including metabolic strain, proteostatic burden, chronic immune activation, and cellular senescence. Senescence-associated cellular dysfunction and pro-inflammatory secretory phenotypes may further amplify chronic stress and diminish systemic resilience in aging neural tissues. Under aging and cumulative stress, progressive loss of buffering capacity leads to resilience exhaustion. Neurodegeneration emerges once critical thresholds are exceeded, reflecting systemic collapse rather than a discrete molecular lesion.

Lower Panel (Biological Constraint-Aware Therapeutic Strategies): This framework emphasizes a shift from late-stage pathology removal toward preservation and modulation of systemic resilience across disease stages. In the early (presymptomatic or preclinical) phase, when compensatory and homeostatic mechanisms remain largely intact, therapeutic strategies focus on tuning excessive stress-response and immune programs through proportional gain modulation rather than binary on/off suppression. In the mid (early symptomatic or transitional) phase, when resilience begins to decline but partial reversibility may remain, strategies aim at rebalancing interdependent systems, including proteostatic flux, inflammatory resolution, and neuron–astrocyte metabolic support. In the late (advanced or decompensated) phase, characterized by substantial loss of resilience and structural damage, therapeutic strategies prioritize buffering and strengthening residual reserve capacity to extend functional viability under fixed constraints. Created in https://BioRender.com.

## Data Availability

No datasets were generated or analysed during the current study.

## Abbreviations

ALS: Amyotrophic lateral sclerosis
APOE ε4: Apolipoprotein E ε4 allele
AP: Antagonistic pleiotropy
NDDs: Neurodegenerative diseases
PQC: Protein quality control
SBMA: Spinal and bulbar muscular atrophy
SPMs: Specialized pro-resolving mediators
UPR: Unfolded protein response

## Declarations

### Authors’ information

S.H.K is a clinician–scientist specializing in neurodegenerative diseases, with research interests focused on evolutionary biology, microglial regulation, and resilience-based therapeutic strategies. M.Y.N is a translational basic scientist specializing in neurodegenerative diseases, with research interests focused on microglial biology, RNA dysregulation, and biomarker-driven mechanistic studies using patient-derived cellular models, aimed at understanding disease heterogeneity and resilience-based therapeutic strategies. M.C.K is a world-leading clinician-scientist specializing in amyotrophic lateral sclerosis (ALS) and clinical neurophysiology. His research interests focus on mechanisms of neurodegeneration, clinical trial design, and the development of novel diagnostic and prognostic biomarkers to improve patient outcomes in motor system disorders.
